# Dynamic changes of SARS-CoV-2 specific IgM and IgG among population vaccinated with COVID-19 vaccine

**DOI:** 10.1017/S0950268822000632

**Published:** 2022-04-08

**Authors:** Fengling Chen, Yi Zhong, Jiazhao Li, Jianrong Luo

**Affiliations:** 1 Department of Laboratory Medicine, Medical Center Hospital of Qionglai City, Chengdu 611530, Sichuan, China; 2 Department of Laboratory Medicine, Qionglai Maternal & Child Health Care Hospital, Chengdu 611530, Sichuan, China

**Keywords:** SARS-Cov-2, COVID-19, IgG, IgM, Vaccine

## Abstract

To evaluate the dynamic changes of antibody levels in different groups after inoculation with the coronavirus disease 2019 (COVID-19) vaccine. The 1493 subjects who were tested for IgM and IgG against severe acute respiratory syndrome coronavirus 2 (SARS-CoV-2) at Qionglai Medical Center Hospital from June to October in 2021 were accepted for analyses of geometric mean titre (GMT) of IgG and IgM. The overall GMT of IgM and IgG in the population of Qionglai reached at a peak value at 1.497 (+3.810, −3.810) S/CO and 4.048 (+2.059, −2.059) S/CO in the second week, and then gradually decreased to 0.114 (+2.707, −2.707) and 1.885 (+1.506, −1.506) S/CO in the 11th–25th weeks, respectively. IgG was positive within 1 day, after that GMT increased continuously and peaked on the 13th day. There was a significant difference between male and female groups for titre of IgM during the prior 2 weeks and among three age groups for titre of IgG during the 2nd–3rd week after vaccination. The GMT level of IgG in the population vaccinated with the COVID-19 vaccine remained at a high level within 25 weeks and peaked on the 13th day, indicating that IgG could exist for a longer period and exhibiting positive SARS-CoV-2- defending effect.

## Introduction

Severe acute respiratory syndrome coronavirus 2 (SARS-Cov-2), a single-stranded sense RNA virus, has infected more than 430 million people worldwide, including more than 5.9 million related deaths (https://covid19.who.int/). The vaccination is one of the powerful measures to prevent and block the transmission of communicable diseases [1], therefore, the coronavirus disease 2019 (COVID-19) vaccine has been recommended for all the countrymen to be administrated to produce the stable and effective antibody to improve the anti-viral capability and reduce the infection rate. There is a dynamic process of antibody level in people after vaccination and the duration of steady antibody is closely related to resistance to infection by that virus. It's worth noting that the population with high risk such as elderly people and immunodeficient individuals at a low level of antibody necessitate more doses of vaccine or monoclonal antibody to enhance antibody production [2, 3]. To date, a total of more than 10 billion vaccine doses have been administered, and the duration of the high level of antibody in serum still needs further observation. According to the COVID-19 Diagnosis and Treatment Protocol released by the National Health Commission of China, SARS-CoV-2 specific antibody detection has been listed as the aetiological evidence for COVID-19 diagnosis together with RNA detection and gene sequencing, and widely used in a clinical laboratory. Besides, it's also a method to monitor the antibody level of people after vaccination. A safe and effective large-scale vaccination is the key intervention measure to control the spread of COVID-19, and antibody detection is a mirror to reflect the effectiveness of the vaccine [4–6]. In this study, the antibody levels of 1093 people who were administrated complete doses of the COVID-19 vaccine were monitored, and to analyse the dynamic changes of IgM and IgG antibodies level in different groups over time.

## Methods

### Study design and subjects

A total of 1493 serum samples from human was collected and detected at Qionglai Medical Center Hospital from June to August 2021, and all people providing samples hadn't been infected with SARS-Cov-2. There were 1218 positive results of antibodies among them with only 1093 people administrated with complete doses of the SARS-CoV-2 vaccine, including 453 males (41.45%), and 640 females (58.55%); 343 cases (31.38%) at 18–45 years old, 528 cases (48.31%) at 46–60 years old, and 222 cases (20.31%) were aged more 60 years, as shown in Table 1. In addition, there were 66 positive results from people with incomplete doses of the vaccine and 59 positive results were from people without administration of the vaccine. To explore this question, 134 negative samples from people who hadn't been vaccinated were collected as control group. There were 275 negative results of antibody, including 105 who administrated with complete doses of vaccine (2 or 3 doses), 36 who administrated with an incomplete dose of vaccine (1 or 2 doses) and 36 who hadn't been administrated vaccine, which had been removed. The time of post-vaccination was calculated from the time of vaccination with the last dose.

**Table 1.** Characteristics of subjects

Time (W)	*n*		*n* (%)						GMT (S/CO)	
		Male	Age groups			Types of vaccines				
			18–45	46–60	>60	Vero cell	CHO cell	Adenovial-vector	IgM	IgG
1	82	43 (52.439)	20 (24.390)	38 (46.341)	24 (29.269)	76 (92.683)	6 (7.317)	0 (0.000)	1.205 (+3.981, −3.981)	1.933 (+3.061, −3.061)
2	207	79 (38.164)	60 (28.986)	101 (48.792)	46 (21.222)	196 (94.686)	10 (4.831)	1 (0.483)	1.497 (+3.810, −3.810)	4.048 (+2.059, −2.059)
3	209	89 (42.584)	62 (29.665)	92 (44.019)	55 (26.316)	204 (97.608)	3 (1.435)	2 (0.957)	0.906 (+4.505, −4.505)	3.526 (+1.890, −1.890)^#^
4	152	62 (40.789)	39 (25.658)	86 (56.579)	27 (17.763)	147 (96.711)	3 (1.974)	2 (1.315)	0.567 (+4.306, −4.306)*	3.192 (+1.906, −1.906)^#^
5–6	232	97 (41.810)	82 (35.345)	111 (47.845)	39 (16.810)	229 (98.707)	2 (0.862)	1 (0.431)	0.418 (+3.680, −3.680)*	2.892 (+1.956, −1.956)^#^
7–8	135	53 (39.259)	43 (31.852)	64 (47.407)	28 (20.741)	134 (99.259)	0 (0.000)	1 (0.741)	0.470 (+3.860, −3.860)*	2.477 (+1.807, −1.807)^#^
9–10	38	16 (42.105)	11 (28.947)	23 (60.526)	4 (10.270)	38 (100.000)	0 (0.000)	0 (0.000)	0.237 (+3.965, −3.965)*	2.325 (+1.721, −1.721)^#^
11–25	38	12 (31.579)	25 (65.789)	13 (34.211)	0 (0.000)	38 (100.000)	0 (0.000)	0 (0.000)	0.114 (+2.707, −2.707)*	1.885 (+1.506, −1.506)^#^

*P < 0.05, *vs*. IgM in second week; ^#^*P* < 0.05, *vs*. IgG in second week.

The types of vaccines including: Anhui Zhifei Biologics Co., Ltd. China (CHO cells, 3 doses); Beijing Zhifei Lvzhu Biopharmaceutical Co., Ltd. China (CHO cells, 3 doses); Sonovac Life Science Co., Ltd. Beijing, China (Vero cells, 2 doses); Beijing Institute of Biological Products CO., Ltd. Beijing, China (Vero cells, 2 doses); Beijing Institute of Biological Products CO., Ltd. Chengdu, China (Vero cells, 2 doses); Beijing Institute of Biological Products CO., Ltd. Lanzhou, China (Vero cells, 2 doses); Shenzhen Kangtai Biological Products Co., Ltd. China (Vero cells, 2 doses) and CanSino Biologics Inc. China (Adenovirus vector, 1 dose). And the people were administrated with vaccines from different manufactures having the same vaccine regime and dose. At last, the sample schedule was shown in Supplementary Table 1. Individual consent for this retrospective analysis was waived.

### Instrument and reagent

The 3 ml venous blood was collected in EDTA-K_2_ tube and centrifuged at 3000 r/min for 10 min to separate plasma for detection. The detection instrument is provided by Mindray I3000, China. The SARS-CoV-2 specific IgM and IgG antibody detection kits (magnetic particle chemiluminescence immunoassay) of Chengdu Mike Biological Co., LTD were used for detection.

### Statistical analysis

GraphPad Prism 8 software (GraphPad Software, LLC) and SPSS23.0 software were used for statistical analysis. The continuous measures were described as frequency and percentage (%). The quantitative data in accordance with normal distribution were expressed as mean ± standard deviation (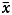 *±* *s*), and quantitative data in accordance with non-normal distribution were expressed as *M* (*P*_25_, *P*_75_), and the geometric mean titre was expressed as *G* (*+* *s.d.*, *−* *s.d.*). All data were compared with the chi-square (*X*^2^) and *t*/*F* test, and the threshold of significance was 0.05. The factors for false-positive results were analysed with a multiple Logistic regression model. The difference was statistically significant at *P* value less than 0.05.

## Results

### Characteristics of subjects

Among the 1218 antibody-positive samples, 59 (data excluded) cases were not vaccinated with the COVID-19 vaccine, and 66 (data excluded) were not vaccinated with complete doses of the COVID-19 vaccine. The age of 1093 people who were vaccinated with complete doses of COVID-19 vaccine ranged from 18 to 86 years old, with a median of 51 years old. There were 1062 samples from people who were vaccinated with inactivated Vero cells based vaccines, 24 inactivated CHO cells based vaccines and 7 adenovial-vector-based vaccines. The other characteristics of the subjects are shown in Table 1.

### Dynamic changes of antibody titre

GMT (S/CO) of IgM from people who were administrated with complete doses of COVID-19 vaccine reached at peak value at the second week, and then the IgM level gradually decreased over time, turning negative (GMT < 1 S/CO) at the third week (*P* < 0.05). GMT (S/CO) of IgG turned positive (GMT ≥ 1 S/CO) in the first week, and reached at peak value at 1.933 (+3.061, −3.061) in the second week, and then the IgG level gradually decreased with time similarly (*P* < 0.05), but remained at a high level at 1.885 (+1.506, −1.506) S/CO without turning negative during 25-weeks observation period, as shown in Table 1. In addition, IgM was negative during 6 days after inoculation and turned positive at 1.626 (+3.098, −3.098) on the 7th day, and peaked at 2.516 (+3.739, −3.739) on the 8th day. IgG was positive at 1.162 (+2.359, −2.359) within a short time after inoculation (within 1 day), and then GMT increased continuously and peaked at 5.291 (+1.498, −1.498) on the 13th day (*P* < 0.05), as shown in Table 2. In addition, there was no significant difference about GMT between two groups with inactivated vaccine type of Vero cells and CHO cells in 1, 2 and 3–4 weeks (*P* > 0.05), respectively. Nevertheless, there was no enough samples to make statistical analysis about groups with adenovial-vector-based vaccines and inactivated vaccines, as shown in Table 1.

**Table 2.** GMT level of SARS-Cov-2 specific IgM and IgG at different time during 14 days

Time (D)	IgM (S/CO)	IgG (S/CO)	Time (D)	IgM (S/CO)	IgG (S/CO)
1	0.370 (+1.914, −1.914)	1.162 (+2.359, −2.359)	8	2.516 (+3.739, −3.739)	2.551 (+1.846, −1.846)*
2	0.257 (+3.872, −3.872)	1.452 (+2.410, −2.410)	9	1.144 (+4.043, −4.043)	3.846 (+1.630, −1.630)
3	0.896 (+2.536, −2.536)	1.917 (+1.653, −1.653)	10	2.128 (+3.560, −3.560)	4.784 (+3.757, −3.757)
4	0.858 (+7.026, −7.026)	1.480 (+2.351, −2.351)*	11	1.228 (+3.634, −3.634)	2.498 (+1.974, −1.974)
5	0.369 (+2.531, −2.531)	1.345 (+6.649, −6.649)*	12	1.597 (+4.474, −4.474)	3.890 (+1.543, −1.543)
6	0.788 (+3.398, −3.398)	1.066 (+2.289, −2.289)*	13	1.701 (+4.663, −4.663)	5.291 (+1.498, −1.498)
7	1.626 (+3.098, −3.098)	2.938 (+2.546, −2.546)	14	1.686 (+3.441, −3.441)	4.729 (+2.007, −2.007)

*P < 0.05, *vs*. IgG on 13th day.

### Association of antibody titre with gender

GMT (S/CO) of IgM in male and female groups during the first week after vaccination were 2.062 (+3.257, −3.257) and 0.667 (+3.885, −3.885) (*P* < 0.05), and GMT (S/CO) of IgM at the second week were 2.125 (+3.795, −3.795) and 1.206 (+3.664, −3.664) (*P* < 0.05). GMT (S/CO) of IgM in the 7th–8th week were 0.802 (+4.226, −4.226) and 0.332 (+3.232, −3.232), respectively (*P* < 0.05), but there was no significant difference for GMT (S/CO) of IgM between male and female groups during other periods (*P* > 0.05). GMT (S/CO) of IgG in male and female groups were positive at 2.031 (+3.530, −3.530) and 1.830 (+2.586, −2.586), respectively, but there was no significant difference during different periods (*P* > 0.05) (Table 3).

**Table 3.** GMT level of SARS-Cov-2 specific IgM and IgG between male and female at different time

Time (W)	IgM (S/CO)		*P*	IgG (S/CO)		*P*
	Male	Female		Male	Female	
1	2.062 (+3.257, −3.257)	0.667 (+3.885, −3.885)	<0.05	2.031 (+3.530, −3.530)	1.830 (+2.586, −2.586)	>0.05
2	2.123 (+3.795, −3.795)	1.206 (+3.664, −3.664)	<0.05	3.927 (+2.096, −2.096)	4.124 (+2.041, −2.041)	>0.05
3	1.039 (+4.675, −4.675)	0.818 (+4.370, −4.370)	>0.05	3.396 (+1.967, −1.967)	3.627 (+1.833, −1.833)	>0.05
4	0.717 (+3.969, −3.969)	0.481 (+4.481, −4.481)	>0.05	2.975 (+1.854, −1.854)	3.352 (+1.941, −1.941)	>0.05
5–6	0.579 (+3.732, −3.732)	0.331 (+3.475, −3.475)	>0.05	2.648 (+2.230, −2.230)	3.080 (+1.738, −1.738)	>0.05
7–8	0.802 (+4.226, −4.226)	0.332 (+3.232, −3.232)	<0.05	2.554 (+1.867, −1.867)	2.429 (+1.773, −1.773)	>0.05
9–10	0.303 (+3.031, −3.031)	0.198 (+4.684, −4.684)	>0.05	1.900 (+1.905, −1.905)	2.693 (+1.508, −1.508)	>0.05
11–25	0.137 (+3.650, −3.650)	0.104 (+2.316, −2.316)	>0.05	2.134 (+1.511, −1.511)	1.780 (+1.496, −1.511)	>0.05

### Association of antibody titre with age

GMT (S/CO) of IgG of all age groups in the second week after inoculation were 4.968 (+1.609, −1.609) (18–45 years old), 3.746 (+2.103, −2.103) (46–60 years old), 3.675 (+2.423, −2.423) (>60 years old) (*P* < 0.05), and IgG in the third week were 4.091 (+1.841, −1.841) (18–45 years old), 3.627 (+1.923, −1.923) (46–60 years old), 2.833 (+1.796, −1.796) (>60 years old) (*P* < 0.05), at last there was no significant difference in GMT (S/CO) of IgG in other age groups (*P* > 0.05). GMT (S/CO) of IgM in different age groups showed no significant difference during different periods (*P* > 0.05) (Table 4).

**Table 4.** GMT level of SARS-Cov-2 specific IgM and IgG in 3-year age groups at different time

Time (W)	IgG (S/CO)				IgM (S/CO)			
	18–45	46–60	>60	*P*	18–45	46–60	>60	*P*
1	2.631 (+2.048, −2.048)	1.704 (+3.920, −3.920)	1.825 (+2.538, −2.538)	>0.05	0.718 (+3.421, −3.421)	1.238 (+3.587, −3.587)	1.778 (+4.791, −4.791)	>0.05
2	4.968 (+1.609, −1.609)	3.746 (+2.103, −2.103)	3.675 (+2.423, −2.423)	0.023	1.271 (+3.612, −3.612)	1.442 (+4.182, −4.182)	2.013 (+3.197, −3.197)	>0.05
3	4.091 (+1.841, −1.841)	3.627 (+1.923, −1.923)	2.833 (+1.796, −1.796)	0.002	0.862 (+4.421, −4.421)	0.960 (+4.605, −4.605)	0.871 (+4.542, −4.542)	>0.05
4	3.329 (+1.790, −1.790)	3.334 (+1.963, −1.963)	2.593 (+1.857, −1.857)	>0.05	0.496 (+4.819, −4.819)	0.650 (+4.047, −4.047)	0.439 (+4.447, −4.447)	>0.05
5–6	2.961 (+2.004, −2.004)	2.985 (+1.907, −1.907)	2.510 (+1.993, −1.993)	>0.05	0.334 (+3.559, −3.559)	0.484 (+3.690, −3.690)	0.440 (+3.791, −3.791)	>0.05
7–8	2.853 (+1.643, −1.643)	2.521 (+1.864, −1.864)	1.916 (+1.802, −1.802)	>0.05	0.421 (+3.915, −3.915)	0.490 (+4.065, −4.065)	0.505 (+3.457, −3.457)	>0.05
9–10	2.558 (+1.568, −1.568)	2.086 (+1.824, −1.824)	3.334 (+1.102, −1.102)	>0.05	0.103 (+4.081, −4.081)	0.359 (+3.591, −3.591)	0.218 (+2.626, −2.626)	>0.05
11–25	1.859 (+1.421, −1.421)	1.935 (+1.679, −1.679)	–	>0.05	0.101 (+2.582, −2.582)	0.144 (+2.946, −2.946)	–	>0.05

### Factors about false-positive results

There were 59 false-positive cases, including 15 IgM (+) IgG (+), 2 IgM (+) IgG (−), and 42 IgM (−) IgG (+). Univariate analysis showed that the increased levels of HBeAb (*P* = 0.005) and HBcAb (*P* = 0.008) were associated with false-positive detection of the SARS-Cov-2 IgG antibody possibly. Multivariate analysis showed that HBeAb (OR = 1.015, *95%CI* 1.001–1.028) maybe a factor causing the false-positive result of the SARS-Cov-2 IgG antibody (*P* = 0.035), as shown in Table 5.

**Table 5.** Analyses of factors about false positive result

Variables	False positive group	Control group	Univariate analyse		Multivariate analyses	
			*X*^2^/*t*	*P*	OR (*95%CI*)	*P*
Gender	Male (36, 61.017%)	Male (75, 55.970%)	0.145	0.704	–	–
Age	47.356 ± 17.191	50.164 ± 25.127	0.903	0.368	–	–
Hepatitis B surface antibody (HBsAb)	6.560 (3.460, 95.540)	7.190 (2.570, 75.623)	1.026	0.309	–	–
Hepatitis B e antibody (HBeAb)	1.410 (1.245, 4.455)	1.260 (1.150, 1.478)	2.89	0.005	1.015 (1.001,1.028)	0.035
Hepatitis B core antibody (HBcAb)	10.860 (4.250, 74.065)	4.835 (4.245, 11.800)	2.711	0.008	1.004 (0.995,1.013)	0.386
Thyroid peroxidase antibody (TPOAb)	4.300 (2.250, 13.350)	4.900 (2.600, 19.900)	0.62	0.538	–	–
Thyroglobulin antibody (TGAb)	7.035 (5.290, 11.585)	7.610 (5.660, 9.625)	1.202	0.26	–	–
Thyrotropin receptor antibody (TRAb)	14.650 (10.380, 23.868)	11.420 (10.633, 13.380)	0.948	0.347	–	–
C-reactive protein (CRP)	0.800 ± 0.001	0.813 ± 0.082	0.634	0.527	–	–
Rheumatoid factor (RF)	9.400 (8.350, 11.700)	9.800 (8.350, 16.100)	0.067	0.947	–	–
Anti-streptococcal lysozyme O (ASO)	33.000 (15.000, 44.000)	14.000 (4.000, 49.000)	0.389	0.699	–	–

## Discussion

Previous studies have revealed that those who could generate the immune response after vaccination (IgM/IgG can be detected in vivo) may still be infected with SARS-Cov-2. Nevertheless, they can rapidly produce a large number of IgG that perform a protective function after infection in comparison to those who had not been vaccinated [7]. The National Health Committee of China had issued the ‘COVID-19 survivors recovery plasma treatment (trial edition)’ to utilise the plasma of COVID-19 survivors as a therapeutic regimen, which indicates that it exerts important effects for COVID-19 patients with the plasma antibody at a high titre. Generally, the serum antibody level is one of the important indicators to evaluate the risk of epidemic disease, but the protective concentration of COVID-19 antibody is unknown at present. One of the possible reasons that those vaccinated and having antibodies against the COVID-19 vaccine were infected by SARS-Cov-2 is that some IgG are not neutralising antibodies and play a limited protective role [8]. Second, there may be an immune escape reaction because of the mutation of the virus [9]. Third, the protective titre of antibody gradually attenuates at a lower level than the protective level to be subject to the virus. This requires further neutralising antibody test to verify the effectiveness of the antibodies generated by vaccination.

IgM is secreted at first in the early stage of infection with pathogens, but it exists for a short time and can be used as an indicator of acute infection. IgG is generated later than IgM, but it can exist for a long periods, which can be used as an indicator at mid- to late-infection or past infection. Prior studies have shown that the titres of IgM and IgG in patients who were infected with SARS-Cov-2 presented the trends of increasing first and decreasing later [10]. In this study, IgG is generated rapidly in the first week after vaccination (on the first day), and the proportion of IgM (−) IgG (+) in the antibody-positive population is 82.92%. The antibody titre reaches the peak value on the 13th day, after that gradually decreases, but still remained at a high level and the proportion increases to 100.0%. IgM turns positive on the 7th day after inoculation, and then the antibody titre gradually decreases, and turns negative at the third week. Although this change curve is inconsistent with the trend of antibody in patients who are infected with SARS-Cov-2 for the first time [7], there is no conflict between them. In this study, there were 7 cases (0.64%) administrated with 1 dose of adenovirus vector-based vaccine, 1062 cases (97.16%) administrated with 2 doses of Vero cell-based vaccine, and 24 cases (2.2%) administrated with 3 doses of CHO cell-based vaccine. Therefore, 99.36% of the population had been vaccinated before the last dose. On the one hand, IgG antibody was still present in their bodies possibly and IgM had turned negative. On the other hand, IgG could be secreted rapidly in vivo after the second or third administration, which are the reasons that IgG is positive on the first day after vaccination. In addition, there was no significant difference between people with two types of vaccine-based Vero and CHO cells, and the size of samples of adenovirus vector-based vaccine was too small to make influence to database in this study.

The existence period of effective IgG in people who had recovered from COVID-19 is 3–8 weeks approximately [11, 12]. In this study, IgG could stabilise at a high titre for 1–2 weeks after COVID-19 vaccine inoculation, subsequently begins to decline slowly, and it exists during 10–25 weeks. Although the decline amplitude is not significant, it's uncertain whether the antibody function is affected. Therefore, SARS-COV-2 may still be at risk of transmission under universal vaccine coverage, and strong public health interventions measures are still required for the prevention.

IgM could be measured in the 3rd–5th day after infection with SARS-Cov-2, IgG could be detected in the 10th–15th day [13, 14]. In this study, IgM begins to exist on the 7th day after vaccination, which is slightly later than that after natural infection. IgG already exists at the time of the second vaccination, hence this population who were administrated with only first dose of vaccine were observed in this study. It is worth noting that IgG would turn positive on the second day that is earlier than the time after natural infection of virus, however, more samples need to be analysed to identify it. In the course of the first 2 weeks after vaccination, IgM level in males is significantly higher than that in females, which is caused by the difference of immune response intensity from a different gender. Subsequently, the antibody titre begins to decline and there is no significant difference between antibody levels in males and females after the third week. There is no significant difference in IgG levels between male and female groups, which reach the peak in the second week and then gradually decreases without a significant difference about the magnitude of the decrease. There is no significant difference among IgM levels in different age groups, which gradually decreases and turns negative in the third week. The peak of IgG is higher from the young group (18–45 years old) at 4.968 (+1.609, −1.609) S/CO and existed for a longer time (about 2 weeks), while the elderly group (>60 years old) has a lower peak of IgG level at 3.675 (+2.423, −2.423) S/CO and a shorter time (about 1 week), which may be associated with the depression of immunity in the elderly.

The rheumatoid factors, autoantibodies and other substances in the body can interfere with detection results and cause false-positive result for specific COVID-19 antibody detection [15]. In this study, 59 of the 1218 cases who presented positive results about COVID-19 antibody are not vaccinated with the COVID-19 vaccine, and the false positive rate is 4.84% and IgG false positive rate is 96.61% in them. In order to explore possible interfering factors, 134 patients who hadn't been vaccinated with the COVID-19 vaccine are collected and tested as a control group with negative results, and shows that the high level of HBeAb may be one of the factors causing the false-positive result of COVID-19 antibody test by univariate and multivariate logistic regression analysis.

There are still quite a few deficiencies in this study. For example, the number of samples after 10 weeks with vaccination of COVID-19 vaccine is relatively small, which is limited to reflect the real condition. The study population is limited to the Qionglai area, while the differences of area, types of vaccination and time between two vaccination are factors that could affect the antibody titre level. Further studies are required to be developed through expanding the scope and sample size. In addition, there is no randomisation of the vaccinated population to determine the antibody-positive rate after vaccination. The antibody titre in this study can not represent the neutralising antibody titre, and the evaluation about the antiviral effect is limited, which requires further neutralisation antibody experiments to prove.

In conclusion, the titre level of IgG of people receiving administration of COVID-19 vaccine in the Qionglai area reaches at the peak on the 13th day and then gradually decreases over time, but it is still at relatively a high level within 25 weeks, suggesting that IgG can exist in a long-term and stable state and exerts good epidemic prevention in the population receiving COVID-19 vaccine. Therefore, vaccination is one of the effective measures to prevent the transmission of SARS-Cov-2. The dynamic changes of antibody can also provide a theoretical basis about boosting vaccination in special population.

## Data Availability

The data is available. The data that support the findings of this study are described in Supplementary Table 1.

## Supplementary material

For supplementary material accompanying this paper visit https://doi.org/10.1017/S0950268822000632. click here to view supplementary material
